# Identifying Obstructive Sleep Apnea in At-Risk Youth: An Exploratory Cross-Sectional Study in Adolescents Living With Obesity

**DOI:** 10.33549/physiolres.935752

**Published:** 2025-12-01

**Authors:** Milos CHUDY, Nikol GOTTFRIEDOVA, Adela NOVOTNA, Petra BLAZKOVA, Bohumila LOKAJOVA, Radovan BUNGANIC, Jana SLONKOVA, Jan BOZENSKY, Marek BUZGA

**Affiliations:** 1 Department of Epidemiology and Public Health, Faculty of Medicine, University of Ostrava, Ostrava, Czech Republic; 2 Department of Physiology and Pathophysiology, Faculty of Medicine, University of Ostrava, Ostrava, Czech Republic; 3 Department of Neurology, University Hospital Ostrava, Ostrava, Czech Republic; 4 Department of Pediatrics, Vitkovice Hospital, Ostrava, Czech Republic; 5 Department of Clinical Neurosciences, Faculty of Medicine, University of Ostrava, Ostrava, Czech Republic; 6 Institute of Laboratory Medicine, University Hospital Ostrava, Ostrava, Czech Republic

**Keywords:** Adolescents living with obesity, Cardiorespiratory polygraphy, Obstructive sleep apnea, Screening, Sleep-disordered breathing

## Abstract

Obstructive sleep apnea (OSA) is one of the most common sleep disorders, affecting 1–10 % of children. A key risk factor is elevated body mass index (BMI). This exploratory study aimed to assess OSA prevalence and severity in adolescents living with obesity and explore associations with clinical and metabolic parameters. Adolescents with obesity aged 10–15 years hospitalized for weight management were enrolled. Participants underwent examination including anthropometry, blood pressure, and lipid profile. BMI was evaluated using WHO BMI-for-age z-scores and an internal standard deviation score (SDS). Sleep-disordered breathing was assessed using cardiorespiratory polygraphy. OSA severity was classified by pediatric AASM criteria using the apnea-hypopnea index (AHI). Among 26 adolescents, OSA occurred in 25 (96.2 %). Median AHI was 9.6 (IQR 5.3–19.1); 44 % had severe, 40 % moderate, and 16 % mild OSA. Severe OSA was more frequently observed in boys (p=0.045), who also showed significantly higher BMI z-score, ODI_3_ and T_90_ values; (p<0.05). Adolescents with severe OSA had higher body weight and BMI z-scores; (p<0.05). In regression models using BMI SDS, male sex emerged as a borderline predictor of higher AHI (β=9.07; p=0.051), while age and BMI metrics were not significant. Spearman analysis further revealed a moderate positive correlation between BMI z-score and T_90_ (ρ=0.51, p=0.02). In this exploratory study, OSA was detected in the majority of adolescents living with obesity, though results should be interpreted with caution. Early recognition may support interventions to limit adverse outcomes. Larger polysomnographic studies with control groups are required to confirm prevalence and clarify risk factors.

## Introduction

Sleep is a fundamental physiological process essential for overall health. Sleep disorders adversely affect multiple organ systems. Poor sleep quality has been linked to serious conditions, including cardiovascular, metabolic, oncological, neurological, psychological, and infectious diseases. It has also been linked to increased all-cause mortality [1,2,3].

Obstructive sleep apnea (OSA) is a relatively common sleep disorder in childhood and adolescence, defined by recurrent episodes of partial or complete collapse of the upper airway during sleep. According to the American Academy of Sleep Medicine (AASM) pediatric guidelines [4], these events are characterized by reduced or absent airflow lasting at least two respiratory cycles, typically accompanied by oxygen desaturation or subsequent arousal. The resulting intermittent hypoxemia and sleep fragmentation are key mechanisms linking OSA to adverse outcomes, including excessive daytime sleepiness, cognitive and behavioral impairment, and increased cardiovascular risk [2,5].

OSA affects approximately 1–10 % of children [6]. However, in children and adolescents with overweight or obesity, prevalence is substantially higher, with rates reported between 24 % and 61 % [7]. The clinical presentation is variable, and symptoms may include growth disturbances, attention and behavioral problems, poor academic performance, and excessive daytime sleepiness. Nocturnal enuresis may also occur in some cases. If left untreated, OSA increases the risk of cardiopulmonary complications and other serious health problems [5,6,8]. Risk factors for pediatric OSA are relatively well established, with adenotonsillar hypertrophy and obesity being the most prominent. However, compared with adults, OSA in children, particularly in adolescents, remains less extensively studied. Early recognition and treatment are essential to reduce long-term complications and improve quality of life [5,9].

The aim of this cross-sectional exploratory study was to determine the prevalence and severity of OSA in adolescents living with obesity and to examine its associations with selected clinical and metabolic parameters. The findings are intended to provide preliminary insights to guide future research.

## Materials and Methods

Single-center, hospital-based cross-sectional exploratory study was conducted at the Department of Pediatrics, Vitkovice Hospital, in collaboration with the Centre for Nutrition Research and Obesitology, Faculty of Medicine, University of Ostrava. Adolescents aged 10–15 years living with obesity, defined as body mass index (BMI) at or above the 95^th^ percentile for age and sex according to WHO standards, were consecutively enrolled during short-term hospitalization for obesity treatment. Recruitment took place from September 2023 to December 2024.

Inclusion criteria were: (1) age 10–15 years; (2) BMI ≥95^th^ percentile for age and sex; (3) completion of overnight cardiorespiratory polygraphy performed during hospitalization; and (4) written informed consent provided by a parent or legal guardian. Exclusion criteria were: (1) incomplete or artefact-dominated polygraphic recordings (defined as loss of airflow or oximetry signal for ≥50 % of the recording); and (2) previous diagnosis of OSA or other sleep disorder. Of 33 enrolled patients, 7 (21 %) were excluded due to excessive artifacts, leaving 26 complete datasets for analysis.

### Data collection and processing

During hospitalization, all participants underwent a standardized clinical assessment, including demographic characteristics, anthropometry, and blood pressure measurement. BMI was calculated as kg/m^2^ and expressed as WHO BMI-for-age z-scores as well as an internal BMI standard deviation score (SDS).

Laboratory testing comprised a lipid profile obtained during hospitalization. Serum triglycerides, total cholesterol, high-density lipoprotein (HDL) cholesterol, and low-density lipoprotein (LDL) cholesterol were analyzed on a Cobas 6000 analyzer (ROCHE s.r.o., Diagnostics Division) using manufacturer-recommended methods. Results were interpreted with respect to the laboratory’s pediatric reference intervals.

Sleep monitoring was performed during hospitalization using cardiorespiratory polygraphy (PCR) (Samoa lite, Löwenstein Medical SE & Co. KG), a routinely used diagnostic tool for sleep-disordered breathing [10]. Recordings were obtained overnight during sleep, typically lasting 6–8 h. Only patients with recordings fulfilling predefined quality criteria (≥4 h of evaluable signal including airflow and oximetry, <50 % artefact contamination) were included in the analysis. Sleep recordings of our patients were evaluated under the supervision of an experienced neurology-certified sleep specialist.

The leading indicator of the severity of sleep apnea was the apnea-hypopnea index (AHI), defined as the number of apneas and hypopneas per hour of sleep. All patients were evaluated according to pediatric AASM criteria. Respiratory events were scored accordingly: Apnea was defined as a reduction of at least 90 % in the nasal airflow signal lasting at least the duration of 2 breaths during baseline breathing despite respiratory effort. Hypopnea was defined as a reduction in airflow of at least 30 % for the duration of at least 2 breaths with either an arousal or a 3 % decrease in oxygen saturation. Oxygen desaturations of 3 % or more are expressed by the oxygen desaturation index (ODI_3_), which represents the number of desaturation events per hour of sleep. The T_90_ parameter represents the percentage of total sleep time during which a patient’s blood oxygen saturation (SpO_2_) drops below 90 %. An AHI value <1 was considered normal (no evidence of sleep apnea syndrome); values from 1 to <5 indicated mild sleep apnea syndrome, values from 5 to <10 moderate sleep apnea syndrome, and values ≥10 severe sleep apnea syndrome. The obstructive character of apneic events was determined based on the presence of respiratory effort during the episodes [4,5].

The study was carried out in accordance with the Declaration of Helsinki [11]. Before participation, all subjects and their legal representatives (that is, parents or guardians) were informed of the study procedures and provided written consent for the processing of the collected data. All participants and their legal representatives had the opportunity to ask questions and withdraw from the study at any time without providing a reason.

### Statistical analysis

Descriptive statistics are presented as median with interquartile range (IQR) for all variables to ensure clarity and consistency. Normality of distribution was assessed using the Shapiro-Wilk test, and group comparisons were performed using the Student’s *t*-test or the Mann-Whitney U test, as appropriate. For clarity and consistency, descriptive data are presented uniformly. Associations between categorical variables were evaluated using Fisher’s exact test.

Correlations between continuous variables were examined using Spearman’s rank correlation coefficient, with adjustment for multiple testing performed by controlling the false discovery rate (FDR) according to the Benjamini-Hochberg procedure.

To evaluate potential predictors of the apnea-hypopnea index (AHI), we applied multivariable regression models. Both ordinary least squares (OLS) regression with heteroskedasticity-robust (HC3) standard errors and median regression were fitted, including age, sex, and BMI z-scores/BMI SDS as covariates. Analyses were conducted in Stata v.13. Data were tested at the significance level of 5 %.

### Ethics declaration

This study was approved by the Ethical Committee of AGEL Hospital Ostrava-Vítkovice (reference number: EK/56/2023).

## Results

The final study cohort included 26 adolescents (14 girls, 53.8 %) with a median age of 13.0 years (IQR 12.0–14.0). Obstructive sleep apnea (OSA) was detected in 25 participants (96.2 %). Of these, four (16.0 %) had mild, ten (40.0 %) moderate, and eleven (44.0 %) severe OSA. Severe OSA occurred predominantly in boys (66.7 %), while girls more frequently presented with mild to moderate OSA (71.4 %). The difference was statistically significant comparing severe versus non-severe categories (p=0.045). All demographic and anthropometric, clinical and sleep-related, and laboratory parameters are summarized in Table 1.

### Demographic and anthropometric data

Age and most anthropometric measures did not differ significantly between boys and girls. A significant sex-related difference was observed only in BMI z-scores, which were higher in boys (p=0.032).

According to OSA severity, adolescents with severe OSA had higher body weight (p=0.008) and BMI z-scores (p=0.042) than those with moderate OSA. No other statistically significant anthropometric differences were observed between the groups.

### Clinical and sleep parameters

There were statistically significant sex-related differences in ODI_3_ (p=0.042) and T_90_ (p=0.031), with higher values observed in boys. Other clinical and sleep-related measures, including mean SpO_2_, heart rate, and blood pressure, did not differ significantly between sexes. A clinical trend toward higher AHI in boys was noted, although this difference did not reach statistical significance (p=0.057).

By OSA severity, adolescents with severe OSA demonstrated significantly higher AHI (p<0.001), ODI_3_ (p<0.001), and T_90_ (p=0.026) compared with those with moderate OSA, as expected given that these parameters are integral to the definition of OSA severity. No other clinical or sleep-related parameters showed significant differences between severity groups, although a non-significant trend was observed for SpO_2_ (p=0.07).

### Laboratory data

No significant differences were observed in lipid parameters between boys and girls, nor between the moderate and severe OSA groups (p>0.05). Median values of total cholesterol, HDL cholesterol, LDL choles-terol, and triglycerides were comparable across categories, with overlapping interquartile ranges.

### Predictors of AHI

Two OLS regression models were fitted, with Model A including BMI expressed as WHO z-scores and Model B using internally derived BMI SDS (Table 2). In both models, none of the predictors (age, sex, BMI) reached statistical significance with respect to AHI. In Model B, male sex showed a clinically relevant effect (β=9.07, 95 % CI −0.02 to 18.17, p=0.051). The explanatory power of the models was limited but not negligible, with R^2^ values of 0.30 for Model A and 0.31 for Model B, suggesting that sex, age, and BMI together explain part of the variability in AHI. The strongest predictor within these models was male sex.

Median regression, included for its robustness to potential outliers, yielded comparable results, reinforcing the observed trends while likewise not identifying statistically significant predictors.

### Correlation matrix of clinical, anthropometric, and sleep parameters

**Fig. 1:**
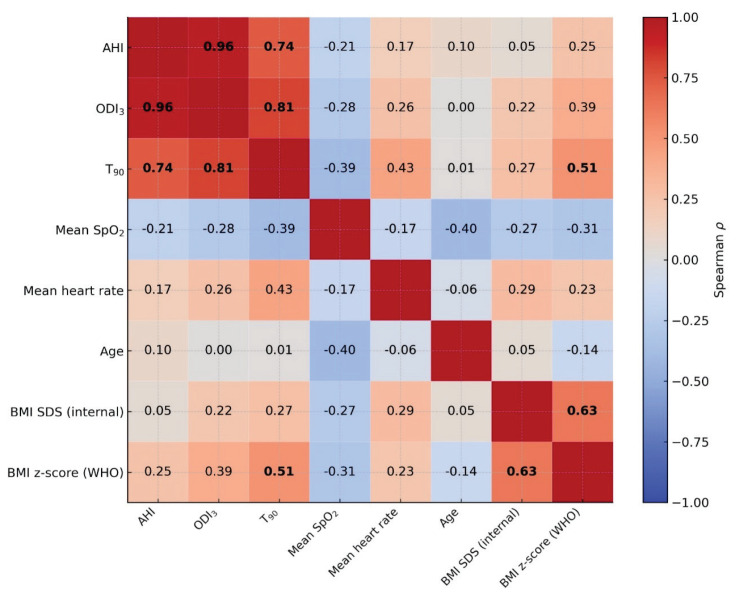
Spearman correlation matrix of anthropometric and sleep parameters with FDR correction. Spearman’s correlation coefficients (ρ) between anthropometric and sleep-related variables. Significant correlations after false discovery rate (FDR) correction (p<0.05) are indicated in bold; AHI – apnea–hypopnea index; ODI_3_ – oxygen desaturation index ≥3 %; T_90_ – percentage of total sleep time with oxygen saturation <90 %; SpO_2_ – peripheral oxygen saturation; BMI SDS – body mass index standard deviation score (internal reference); BMI z-score (WHO) – body mass index z-score according to WHO reference.

Strong and statistically significant correlations were observed between AHI and ODI_3_ (Spearman ρ=0.96), ODI_3_ and T_90_ (Spearman ρ=0.81), and AHI and T_90_ (Spearman ρ=0.74), all of which remained significant after FDR correction (p≤0.001). Additional significant associations were detected between T_90_ and BMI z-score (Spearman ρ=0.51) and between BMI z-score and BMI SDS (Spearman ρ=0.74); (p<0.05). Several other weak-to-moderate correlations were observed, but these were either not statistically significant or lost significance following FDR correction. Mean SpO_2_ showed consistently negative correlations with all examined parameters (Fig. 1).

## Discussion

In our cohort of 26 adolescents living with obesity, we found an exceptionally high prevalence of obstructive sleep apnea (OSA), with 96.2 % of participants meeting diagnostic criteria. Severe OSA was significantly more common in boys compared with girls, and boys also exhibited higher BMI z-scores, ODI_3_, and T_90_ values. Across OSA severity categories, adolescents with severe disease had significantly higher body weight, BMI z-scores, and worse sleep-disordered breathing indices (AHI, ODI_3_, T_90_) compared with those with moderate OSA. No significant differences were observed in lipid parameters between sexes or OSA severity groups. In regression analyses, none of the predictors (age, sex, BMI) reached statistical significance with respect to AHI; however, male sex showed a clinically relevant, near-significant association. Correlation analyses confirmed strong interrelationships between AHI, ODI_3_, and T_90_, and also revealed a moderate positive association between BMI z-scores and T_90_.

In the general pediatric population, OSA prevalence is estimated at approximately 1–6 % [12], with rates up to 10 % also described [6]. Among children and adolescents living with overweight or obesity, the prevalence is substantially higher, ranging between 24 % and 61 % across published studies [7,13]. Andersen *et al*. reported OSA in 44.6 % of obese children compared with 9.1 % in their normal-weight peers, with each one-unit increase in BMI SDS nearly doubling the odds of OSA [7].

In our cohort, OSA was detected in 96.2 % of adolescents hospitalized for obesity management. This proportion far exceeds previously reported prevalence rates in both the general pediatric population and in children living with obesity. Our findings cannot be interpreted as population-based prevalence, but rather as the occurrence within an extremely high-risk subgroup of adolescents with severe obesity. Such an exceptionally high rate should be interpreted with caution and may reflect several limiting factors of our study, including the specific characteristics of the study population (hospitalized adolescents with severe obesity) and the use of polygraphy instead of polysomnography. Although polygraphy is an established diagnostic method, its methodological constraints compared with full polysomnography may in some cases lead to misclassification of OSA severity. In particular, overestimation of the AHI may occur due to frequent artifacts; to minimize this risk, patients with substantial artifacts were excluded from the analysis. Our exploratory study provides preliminary findings that suggest a very high risk of OSA among adolescents with severe obesity and highlight the importance of considering systematic screening in this particularly vulnerable population.

Sex- and age-related differences in the prevalence and severity of pediatric OSA have been described [14]. In prepubertal children, no significant sex differences have generally been observed. The NANOS study reported a comparable prevalence of OSA in obese prepubertal girls (42.5 %) and boys (37 %) [15]. In contrast, during adolescence, OSA tends to be detected more frequently in boys than in girls [7,16]. Selvadurai *et al*. demonstrated a significantly greater occurrence of OSA among obese pubertal males [17], and Kang *et al*. reported that boys had higher AHI values than girls [18]. Overall, current evidence indicates that sex differences in OSA are less pronounced in childhood but may become more apparent during adolescence, with OSA being detected more frequently and at higher severity in boys.

In our cohort, male sex emerged as a clinically relevant predictor of higher AHI, reflecting greater OSA severity. Although the regression models did not reach conventional statistical significance, the effect size for male sex was considerable, suggesting a potential association. Moreover, when stratifying by severity, we observed that severe OSA was significantly more frequently detected in boys, whereas girls were more often classified within the mild-to-moderate categories. These findings are consistent with reports from other pediatric cohorts indicating that male sex becomes a risk factor for more severe OSA during adolescence.

Anthropometric characteristics are important determinants of OSA occurrence and severity in children, with obesity consistently identified as an independent risk factor [19]. Kohler *et al*. demonstrated that this association becomes particularly evident during adolescence: among children aged ≥12 years, each one-unit increase in BMI z-score was associated with a 3.5-fold higher risk of OSA [20]. Consistent with this, Sukharom *et al*. identified a BMI cut-off of 29.2 kg/m^2^ as predictive for severe OSA in obese children, highlighting the importance of specific anthropometric thresholds [12]. In addition, Andersen *et al*. reported that a one-unit increase in BMI SDS nearly doubled the odds of OSA (OR 1.92, 95 % CI 1.33–2.76), independent of age, sex, tonsillar hypertrophy, and asthma [7].

In our cohort, both body weight and BMI z-score were significantly higher in adolescents with severe OSA, while BMI SDS did not differ across severity categories. Regression analyses suggested a positive association of both BMI z-score and BMI SDS with AHI, although statistical significance was not reached. These findings are in line with previous observations that higher BMI may contribute to greater OSA severity in adolescents.

As outlined above, BMI may contribute to higher AHI, and AHI severity is usually accompanied by changes in related respiratory indices such as ODI, T_90_, and mean nocturnal oxygen saturation. Previous studies have suggested that these parameters tend to be more impaired in boys [16,21]. In contrast, Horne *et al*. did not observe significant sex differences in the severity of some sleep-disordered breathing across childhood and adolescence [22]. In our cohort, boys demonstrated significantly worse values of ODI_3_ and T_90_ compared with girls, and T_90_ also showed a moderate correlation with BMI z-score. Mean oxygen saturation did not differ significantly between the examined groups.

OSA, similar to obesity, has been associated with an increased risk of elevated blood pressure in children, although reported findings are not entirely consistent across studies [14,23]. In our cohort, no significant differences in blood pressure were observed between sexes or across OSA severity categories, which may in part reflect the limited sample size. Likewise, no significant differences or associations were detected for mean heart rate, although this parameter may also worsen with increasing OSA severity [24].

In our cohort, no significant differences in lipid parameters were observed between sexes or across OSA severity categories. However, previous studies have reported associations between OSA and dyslipidemia in children. Lei *et al*. demonstrated higher total cholesterol, triglycerides, and LDL-C levels, and lower HDL-C in children with OSA, with triglycerides correlating with BMI z-score and oxygen desaturation [25]. Similarly, Kang *et al*. reported significantly lower HDL-C levels in moderate-to-severe pediatric OSA, even after adjusting for age and BMI [26]. These findings suggest that OSA may contribute to adverse lipid profiles, particularly in obese children.

The occurrence of OSA, which disrupts sleep patterns, should not be underestimated. Early recognition and timely interventions, including treatment approaches that support weight reduction, may not only improve quality of life but also mitigate the risk of long-term complications. Beyond cardiovascular and metabolic consequences, pediatric OSA has been associated with adverse neurocognitive, behavioral, and emotional outcomes, including attention deficits and mood disorders, particularly in adolescents [14]. These findings highlight the importance of prompt diagnosis and comprehensive management of OSA in children and adolescents living with obesity [14,27].

## Limitations

The most important limitation of this study is the small sample size and single-center design, which inherently restrict the statistical power and generalizability of the findings. The study population consisted exclusively of adolescents living with obesity who were hospitalized for weight management. This targeted selection reflects the clinical focus of our work; however, if interpreted as prevalence data, it would introduce a potential selection bias and therefore precludes any extrapolation of our findings to the general adolescent population. The absence of a comparison group of non-obese or less severely obese peers further limits the ability to contrast findings across different weight categories. Nevertheless, the very high detection rate of OSA in this high-risk cohort highlights the relevance of systematic screening in this clinical setting.

Another important limitation is the absence of otorhinolaryngological examination. Although all respiratory events detected by polygraphy were interpreted as obstructive, we did not have systematic data on upper airway anatomy, tonsillar or adenoidal hypertrophy, or other structural conditions that may contribute to OSA in adolescents. Without these data, a proportion of the observed events might have had a different pathophysiological basis, which cannot be fully clarified in our cohort.

In addition, the use of PCR rather than full PSG represents a methodological constraint. PCR is cost-effective, less resource-intensive, and commonly used method, whereas PSG remains the gold standard. However, PCR provides substantially less information than PSG, particularly regarding sleep architecture, REM sleep, and EEG arousals. This methodological choice may therefore misclassify OSA severity compared with the gold standard PSG. Nevertheless, polygraphy remains an established and indispensable tool in the diagnostic work-up of sleep apnea.

## Conclusions

Within our cohort of adolescents living with obesity, OSA was identified in the vast majority of participants (96.2 %). This observation suggests that OSA may represent a common and clinically relevant comorbidity in this high-risk population. Despite the limitations of our study, these findings highlight the importance of early recognition, which could enable interventions aimed at reducing the cardiovascular, metabolic, and neurocognitive risks associated with OSA and obesity. Further research is required to confirm the high occurrence of OSA in this vulnerable group, to better characterize clinical and anthropometric predictors, and to clarify the long-term consequences of pediatric OSA.

## Figures and Tables

**Table 1:** Demographic, anthropometric, clinical, and laboratory characteristics of the study cohort stratified by sex and OSA severity.

*Characteristic*		Sex (n=26)		OSA severity (n=25; 96.2 %)		
		Boys (median, IQR)	Girls (median, IQR)	Mild OSA (median, IQR)	Moderate OSA (median, IQR)	Severe OSA (median, IQR)
*Demographic and anthropometric*	*Age [years]* ^b^	12.50 (11.00–13.25)	13.50 (13.00–14.00)	14.50 (13.75–15.00)	12.00 (11.00–13.75)	13.00 (12.50–14.00)
	*Height [cm]* ^a^	167.00 (161.75–173.00)	159.50 (158.00–164.75)	159.00 (158.75–162.50)	161.50 (158.50–165.00)	168.00 (160.50–173.00)
	*Weight [kg]* ^a^	99.80 (81.00–120.05)	90.90 (80.00–97.30)	93.90 (89.80–100.00)	84.00 (75.47–95.22)	117.80 (89.80–120.70)▪
	*BMI z-score (WHO)* ^a^	3.40 (3.17–3.96)♂	3.19 (2.80–3.35)	3.19 (3.07–3.33)	3.00 (2.80–3.27)	3.51 (3.26–3.96)▪
	*BMI SDS (internal)* ^a^	−0.13 (−0.71–0.71)	0.04 (−0.36–0.61)	0.10 (−0.12–0.26)	−0.24 (−0.71–0.00)	0.71 (−0.48–0.82)

*Clinical*	*AHI [events/h]* ^b^	14.70 (8.60–24.02)	6.80 (4.95–9.67)	4.40 (3.30–4.83)	7.20 (6.08–9.40)	21.80 (16.30–26.10)▪
	*ODI* *_3_* * [events/h]* ^b^	18.00 (12.88–30.85)♂	9.60 (6.15–14.93)	5.10 (3.75–5.85)	9.90 (8.80–11.90)	26.70 (21.00–31.10)▪
	*Mean SpO* *_2_* * [%]* ^a^	96.25 (95.38–96.55)	96.35 (95.53–97.40)	95.65 (95.30–96.60)	96.70 (96.43–97.55)	96.20 (95.05–96.40)
	*T* *_90_* * [%]* ^b^	0.25 (0.10–0.55)♂	0.00 (0.00–0.18)	0.00 (0.00–0.00)	0.10 (0.00–0.18)	0.30 (0.15–0.70)▪
	*Mean heart rate [bpm]* ^a^	72.50 (61.50–77.75)	71.50 (61.50–77.00)	74.50 (68.00–77.00)	67.00 (61.25–76.50)	73.00 (63.50–78.50)
	*Systolic BP [mmHg]* ^a^	120.50 (114.75–124.50)	123.50 (115.00–125.00)	125.50 (124.75–128.00)	117.50 (111.00–125.00)	121.00 (114.50–123.50)
	*Diastolic BP [mmHg]* ^a^	80.50 (75.00–82.25)	81.00 (75.00–84.25)	80.00 (76.75–83.00)	81.00 (75.00–81.75)	80.00 (74.00–82.50)

*Laboratory*	*Total cholesterol [mmol/l]* ^a^	3.54 (3.29–4.29)	3.94 (3.48–4.42)	3.92 (3.41–4.48)	3.91 (3.33–4.97)	3.68 (3.33–4.08)
	*HDL cholesterol [mmol/l]* ^a^	1.11 (0.92–1.28)	1.11 (0.98–1.19)	1.12 (0.99–1.23)	0.99 (0.94–1.12)	1.16 (1.11–1.29)
	*LDL cholesterol [mmol/l]* ^a^	2.17 (2.06–3.02)	2.49 (2.09–3.17)	2.69 (2.09–3.31)	2.55 (2.22–3.49)	2.14 (1.98–2.76)
	*Triglycerides [mmol/l]* ^a^	1.10 (0.95–1.53)	1.58 (0.91–1.71)	1.14 (0.70–1.59)	1.57 (0.90–1.91)	1.14 (1.03–1.54)

Values are presented as median (interquartile range, IQR). OSA = obstructive sleep apnea; BMI = body mass index; SDS = standard deviation score; AHI = apnea–hypopnea index; ODI_3_ = oxygen desaturation index (≥3 % desaturations per hour of sleep); SpO_2_ = peripheral oxygen saturation; T_90_ = percentage of total sleep time with oxygen saturation <90 %; BP = blood pressure; HDL = high-density lipoprotein cholesterol; LDL = low-density lipoprotein cholesterol.

♂ indicates significant sex-related differences;

▪ indicates significant differences between moderate and severe OSA groups (Student’s *t*-test^a^ or Mann-Whitney U test^b^).

**Table 2:** OLS regression models predicting apnea-hypopnea index (AHI).

*Model A* *Predictor*	β (Beta)	SE	95 % CI	p-value
*Intercept*	−24.05	19.33	−61.94 to 13.84	0.214
*BMI z-score (WHO)*	1.54	2.43	−3.23 to 6.31	0.526
*Age [years]*	2.12	1.33	−0.49 to 4.74	0.112
*Male sex*	8.29	5.49	−2.47 to 19.06	0.131

*Model B* *Predictor*	**β (Beta)**	**SE**	**95 % CI**	**p-value**

*Intercept*	−11.42	21.08	−52.74 to 29.90	0.588
*BMI SDS (internal)*	1.76	2.27	−2.69 to 6.20	0.438
*Age [years]*	1.55	1.59	−1.57 to 4.66	0.330
*Male sex*	9.07	4.64	−0.02 to 18.17	0.051

Model A includes BMI expressed as WHO BMI z-scores; Model B uses internally derived BMI SDS. β = regression coefficient; SE = standard error; CI = confidence interval.

## Abbreviations

AHI: apnea-hypopnea index
BP: blood pressure
BMI: body mass index
HDL: high-density lipoprotein
LDL: low-density lipoprotein
ODI: oxygen desaturation index
OSA: obstructive sleep apnea
PCR: cardiorespiratory polygraphy
PSG: polysomnography
SI: snore index
SpO_2_: peripheral oxygen saturation
T_90_: total sleep time with oxygen saturation below 90 %
WHO: World Health Organization

## References

[1] Irwin MR (2015). Why sleep is important for health: a psychoneuroimmunology perspective. Annu Rev Psychol.

[2] Pavlova M, Latreille V (2019). Sleep disorders. Am J Med.

[3] Taylor DJ, Lichstein KL, Durrence HH (2003). Insomnia as a health risk factor. Behav Sleep Med.

[4] Berry RB, Budhiraja R, Gottlieb DJ, Gozal D, Iber C, Kapur VK, Marcus CL (2012). Rules for scoring respiratory events in sleep: update of the 2007 AASM manual for the scoring of sleep and associated events: deliberations of the Sleep Apnea Definitions Task Force of the American Academy of Sleep Medicine. J Clin Sleep Med.

[5] Baker M, Scott B, Johnson RF, Mitchell RB (2017). Predictors of obstructive sleep apnea severity in adolescents. JAMA Otolaryngol Head Neck Surg.

[6] Chan J, Edman JC, Koltai PJ (2004). Obstructive sleep apnea in children. Am Fam Physician.

[7] Andersen IG, Holm JC, Homøe P (2019). Obstructive sleep apnea in children and adolescents with and without obesity. Eur Arch Otorhinolaryngol.

[8] Li Z, Celestin J, Lockey RF (2016). Pediatric sleep apnea syndrome: an update. J Allergy Clin Immunol Pract.

[9] Gouthro K, Slowik JM (2024). Pediatric obstructive sleep apnea. StatPearls.

[10] Ferre A, Rahnama K, Vila J, Cambrodi R, Jurado M, Romero O (2013). Cardiorespiratory polygraphy diagnostic accuracy in mild to moderate obstructive sleep apnea hypopnea syndrome (OSAHS). Sleep Med.

[11] World Medical Association WMA Declaration of Helsinki: ethical principles for medical research involving human subjects.

[12] Sukharom R, Tovichien P, Udomittipong K, Tiamduangtawan P, Chotinaiwattarakul W (2025). Polysomnographic features of children with obesity: body mass index predicts severe obstructive sleep apnea in obese children?. Clin Exp Pediatr.

[13] Al-Iede M, Rahal R, Al-Mashaqbeh S, Alajmi M, Al-Rawi H, Alshrouf M, Ahmad FK (2025). Obstructive sleep apnea in overweight and obese children: factors influencing quality of life. Laryngoscope Investig Otolaryngol.

[14] Romero-Peralta S, Rubio C, Castillo-García M, Resano P, Alonso M, Solano-Pérez E, Silgado L (2024). Obstructive sleep apnea in pediatrics and adolescent women: a systematic review of sex-based differences between girls and boys. Children.

[15] Alonso-Álvarez ML, Cordero-Guevara JA, Terán-Santos J, Gonzalez-Martinez M, Jurado-Luque MJ, Corral-Peñafiel J, Duran-Cantolla J (2014). Obstructive sleep apnea in obese community-dwelling children: the NANOS study. Sleep.

[16] Inoshita A, Kasai T, Matsuoka R, Sata N, Shiroshita N, Kawana F, Kato M, Ikeda K (2018). Age-stratified sex differences in polysomnographic findings and pharyngeal morphology among children with obstructive sleep apnea. J Thorac Dis.

[17] Selvadurai S, Voutsas G, Katz SL, Blinder H, Narang I (2022). Evaluating symptoms and polysomnographic findings among male and female children with obesity with and without obstructive sleep apnea. Sleep Med.

[18] Kang KT, Weng WC, Lee PL, Hsu WC (2022). Age- and gender-related characteristics in pediatric obstructive sleep apnea. Pediatr Pulmonol.

[19] Bachrach K, Danis DO, Cohen MB, Levi JR (2021). The relationship between obstructive sleep apnea and pediatric obesity: a nationwide analysis. Ann Otol Rhinol Laryngol.

[20] Kohler MJ, Thormaehlen S, Kennedy JD, Pamula Y, van den Heuvel CJ, Lushington K, Martin AJ (2009). Differences in the association between obesity and obstructive sleep apnea among children and adolescents. J Clin Sleep Med.

[21] Huang G, Wang Q, Chang L, Ye S, Liu J, Lu Y, Li T (2025). Impact of gender, age, and obesity on childhood obstructive sleep apnea: a cross-sectional study of 4,668 children. Nat Sci Sleep.

[22] Horne RSC, Ong C, Weichard A, Nixon GM, Davey MJ (2020). Are there gender differences in the severity and consequences of sleep disordered breathing in children?. Sleep Med.

[23] Narang I, Mathew JL (2012). Childhood obesity and obstructive sleep apnea. J Nutr Metab.

[24] Brockmann PE (2015). Cardiovascular consequences in children with obstructive sleep apnea: is it possible to predict them?. Sleep.

[25] Lei L, Zhang X, Wang B, Lei F, Dai L, Sun X, Zhao Y (2024). Effects of sleep-disordered breathing on serum lipid levels in children: a case-control study. BMC Pediatr.

[26] Kang EK, Jang MJ, Kim KD, Ahn YM (2021). The association of obstructive sleep apnea with dyslipidemia in Korean children and adolescents: a single-center, cross-sectional study. J Clin Sleep Med.

[27] Guta OM, Iaru OE, Vlad RM (2019). Eat less to sleep more - sleep-related disorders in obese children, a healthcare problem. Rom Med J.

